# Repeat cesarean section in subsequent gestation of women from a birth cohort in Brazil

**DOI:** 10.1186/s12978-017-0356-8

**Published:** 2017-08-25

**Authors:** Keila Cristina Mascarello, Alicia Matijasevich, Aluísio J D Barros, Iná S Santos, Eliana Zandonade, Mariângela Freitas Silveira

**Affiliations:** 1 Center of Epidemiological Research, Post-graduate Program in Epidemiology, Federal University of Pelotas, Pelotas, Rio Grande do Sul Brazil; 2 Healthcare Sciences Department, Federal University of Espírito Santo, São Mateus, Espírito Santo Brazil; 3 Department of Preventive Medicine, Medical School, University of São Paulo, São Paulo, São Paulo Brazil; 4 Post-graduate Program in Public Health, Federal University of Espírito Santo, Vitória, Espírito Santo Brazil; 5 Maternal and Child Department, Medical School, Federal University of Pelotas, Pelotas, Rio Grande do Sul Brazil

**Keywords:** Cesarean section, Natural childbirth, Trial of labor

## Abstract

**Background:**

The current literature indicates increasing concern regarding the number of safe cesarean sections which a woman can undergo, mainly in face of the high cesarean section rates, which are growing in Brazil and worldwide. Aimed to describe the prevalence and associated factors of repeat cesarean section in a cohort of Brazilian women who had a cesarean section in the first birth.

**Methods:**

This is a prospective cohort study using data from the 2004 Pelotas Birth Cohort. The sample included 480 women who had their first delivery in 2004, regardless of the form of delivery, and who had a second delivery identified in the cohort’s follow-ups (in 2005, 2006, 2008, and 2010). Descriptive, bivariate and multivariate analyses using Poisson regression with robust error variance were carried out.

**Results:**

Among the women who underwent a cesarean section in their first delivery (49.47%), 87.44% had a second surgical delivery. The risk factors for repeat cesarean section included ages 21–34 (PR 1.67, CI 95% 1.07–2.60), not being seen by SUS (Public Healthcare System) in 2004 (PR 2.27, CI 95% 1.44–3.60), and the number of prenatal medical visits, i.e., women with ten or more visits were at 2.33 times higher risk (CI 95% 1.10–4.96) compared to those who had five or fewer visits**.**

**Conclusions:**

The proportion of cesarean sections both in the first and in the subsequent delivery is quite high. This high rate may compromise the reproductive future of the women who undergo consecutive cesarean sections with possible consequent complications and changes in care policies for pregnant women should be implemented.

**Electronic supplementary material:**

The online version of this article (doi:10.1186/s12978-017-0356-8) contains supplementary material, which is available to authorized users.

## Plain English Summary

The current literature indicates increasing concern regarding the number of safe cesarean sections which a woman can undergo, mainly in face of the high cesarean section rates, which are growing in Brazil and worldwide. Aimed to describe the prevalence and associated factors of repeat cesarean section in a cohort of Brazilian women who had a cesarean section in the first birth. Among the women who underwent a cesarean section in their first delivery, 87.44% had a second surgical delivery. The risk factors for repeat cesarean section included ages 21–34, not being seen by SUS (Public Healthcare System), and the number of prenatal medical visits, i.e., women with ten or more visits were at 2.33 times higher risk, compared to those who had five or fewer visits**.** The proportion of cesarean sections both in the first and in the subsequent delivery is quite high. This high rate may compromise the reproductive future of the women who undergo consecutive cesarean sections with possible consequent complications.

## Background

The current literature indicates increasing concern regarding the number of safe cesarean sections which a woman can undergo, mainly in face of the high cesarean section rates, which are growing in Brazil and worldwide [1–3]. In 2009, the rate of deliveries through cesarean section in Brazil was 50.1%, for the first time surpassing the number of vaginal deliveries. This number continues to increase and cesarean sections represented 55.7% of the deliveries in 2014 [4].

The growing number of cesarean sections leads to a higher number of repeat cesarean sections partially due to the belief that “once a cesarean section, always a cesarean section” [5], which was widely popular in the obstetrical practice in the 20th century and still permeates the routine of a large number of professionals and services.

Despite the practice of repeat cesarean sections in subsequent deliveries, the obstetrical protocols recommend that women with prior cesarean sections with low transverse scar are candidates to vaginal delivery and that they must be informed of that. In case absolute cesarean section indicators are present, the women must undergo trial of labor [6, 7].

The main concern regarding vaginal delivery after previous cesarean section is the greater risk of uterine rupture during labor and delivery [8]. A case-control study carried out in the United Kingdom found an overall estimated uterine rupture rate of 0.2 per 1000, 2.1 per 1000 women with planned vaginal delivery after previous cesarean section and 0.3 per 1000 in elective repeat cesarean sections [9]. The odds of uterine rupture was higher among women with two or more previous cesarean sections, those with a short interval since the last cesarean section, and those who underwent induced delivery. Although the rupture is associated with mortality and morbidity, it is a rare occurrence even in a vaginal delivery after a previous cesarean section [9]. Moreover, two meta-analyzes found a lower risk of other serious complications among women having vaginal delivery after a previous cesarean section, which counterweighs the risks [8, 10].

Despite the existing recommendations, a meta-analysis published in 2010 showed that, in studies started in 1996, fewer than half (44%) of the women actually underwent trial of labor, compared to 62% of the women in studies started prior to 1996 [11].

Therefore, the present study aimed to describe the prevalence and associated factors of repeat cesarean section in a cohort of Brazilian women who had a cesarean section in the first birth.

## Methods

This is a prospective cohort study that uses data from the 2004 Pelotas Birth Cohort. This cohort includes all births that occurred in 2004 by mothers living in the urban area of the city of Pelotas, Rio Grande do Sul, Brazil, and in the Jardim América neighborhood in the neighbor city of Capão do Leão.

The year of 2004 saw 4287 children born in Pelotas. Of those, 4231 were live births and the 4189 mothers (due to multiple births) were invited to take part in the study, which included interviews with the mothers and an evaluation of the neonates. When the children turned three, 12, 24, and 48 months and 6 years old, the mothers were contacted for follow-ups (at home up to 48 months and in a clinic at the Medical School at 6 years) and interviews with standardized questionnaires applied by trained interviewers. The three-month follow-up included 3985 children and their mothers; the 12-month, 3907; the 24-month, 3869; the 48-month, 3799; and the six-year, 3722. The losses and refusals from the beginning of the study to the six-year follow-up added up to 9.8% (414 children). More details on the methodology, including the sample’s characteristics, can be obtained in another publication [12].

The present study used information on the demographic, socioeconomic, and obstetrical characteristics obtained from the perinatal study (2004). The data of subsequent pregnancies were surveyed in the other follow-ups.

The study included only women who had their first child in 2004 (primiparous), regardless of the mode of delivery, since the previous mode of delivery in multiparous women might impact their choice in subsequent deliveries. The outcome was repeat cesarean section among these women, i.e., two consecutive cesarean sections. The other variables included were living with the husband or partner, the asset index (AI) [13], schooling, number of prenatal medical visits, being seen by the Public Healthcare System (SUS) (public payment) or out of pocket and health insurance (private payment) in the birth in 2004, mother’s skin color, and mother’s age. The continuous variables were categorized for the analyses. The AI was categorized into quintiles, for the total sample; schooling, into three categories, i.e., 0–8, 9–11, and 12 or more full years of education; the number of prenatal medical visits was categorized into five or fewer, 6–9, or 10 or more visits; age was categorized into 20 years or less, 21–34 and 35 or older.

The analysis was carried out using the statistical software Stata 13.0. Descriptive analyses on the primiparous, as well as on the characteristics of subsequent deliveries for those who had them, were performed. The association between the characteristics surveyed and the repeat cesarean sections was assessed using Poisson regression with robust error variance. The multivariate analysis included all variables associated with repeat cesarean section at *p* < 0.20 in the bivariate analysis. To check for interaction between two variables and the outcome, when there was theoretical support, the heterogeneity test was used.

The study’s protocol was approved by the Committee of Research Ethics of the Medical School of the Federal University of Pelotas. The subjects signed a term of free and informed consent at each follow-up after clearing their doubts about the research procedures. *It is not trials of health care interventions*.

## Results

**Fig. 1:**
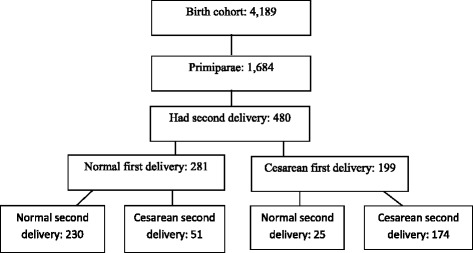
Selection of the subjects included in the study

Among the 4189 mothers of children included in the cohort, 1684 delivered their first child in 2004 and were analyzed in this study (Fig. 1). Table 1 presents the sociodemographic and obstetrical characteristics. A higher number of mothers were in the fifth quintile in the AI classification, revealing that a larger number of primiparous were among the richer levels of the population. Similar results were found for schooling with 56% of the women having nine or more years of education. Regarding the healthcare services variables, 75.65% were seen by SUS during labor at first birth and 14.28% attended five or fewer prenatal medical visits. As for the mode of delivery, cesarean section accounted for 49.47% of all deliveries in the first pregnancy. It was also that episiotomy was performed in 88.62% of the primiparous who underwent vaginal delivery.

**Table 1:** Sociodemographic and obstetrical characteristics of the primiparous mothers

Variável	Number	Percent	Total
Lives with the husband or partner			
No	402	23.87	1684
Yes	1282	76.13	
Schooling			
0–8	731	43.98	1662
9–11	706	42.48	
12 or more	225	13.54	
Age			
<=20	758	45.01	1684
21–34	860	51.07	
35 or more	66	3.92	
Asset index (AI)			
1st quintile	205	16.4	1250
2nd quintile	214	17.12	
3rd quintile	242	19.36	
4th quintile	285	22.8	
5th quintile	304	24.32	
Number of prenatal medical visits			
0–5	231	14.28	1618
6–9	744	45.98	
10 or more	643	39.74	
Seen by SUS^a^			
Yes	1274	75.65	1684
No	410	24.35	
Skin color			
White	1307	77.61	1684
Black	283	16.81	
Other	94	5.58	
Mode of delivery in 2004			
Vaginal	851	50.53	1684
Cesarean section	833	49.47	
Episiotomy			
No	95	11.38	835
Yes	740	88.62	

^a^Public Healthcare System

Most of the primiparous (57.02%) had not been pregnant again until the six-year follow-up. Of the others, 81.77% (480) had already had their second delivery by the last interview, whereas the remaining women were still pregnant or had had a miscarriage. Among the women who underwent a cesarean section in the first delivery, 87.44% had a repeat cesarean, among women with vaginal first delivery, 18.15% underwent a cesarean section in the second delivery.

Table 2 presents the bivariate analysis between repeat cesarean section and the independent variables. In this analysis, the women with higher schooling were at higher risk of repeat cesarean section (PR 2.55, CI 95% 1.98–3.29), as well as the richest ones, i.e., those belonging to the fifth quintile in the AI (PR 2.84, CI 95% 1.82–4.45), and ten or more prenatal medical visits (PR 2.82, CI 95% 1.75–4.56). Regarding the maternal age, women 21–34 and 35 or more were at 2.2 and 2.92 higher risk of undergoing a repeat cesarean section compared to those below 20 years old. The women who were not seen by SUS were at 2.87 times higher risk of having two consecutive cesarean sections compared to those seen by SUS (CI 95% 2.34–3.54). Skin color and living with the husband or partner were not related to repeat cesarean sections.

**Table 2:** Raw and adjusted prevalence ratios (PR) for repeat cesarean section in a subsequent pregnancy

	Repeat cesarean section		Raw analysis			Adjusted analysis		
	No	Yes	PR	CI 95%*	*p*-value	PR	CI 95%*	*p*-value
Schooling								
0–8	178 (71.77)	70 (28.23)	1	-	**0.001**	1	-	0.061
9–11	107 (66.46)	54 (33.54)	1.1	0.88–1.59		0.61	0.39–0.95	
12 or more	17 (27.87)	44 (72.13)	**2.55**	**1.98–3.29**		0.76	0.46–1.27	
Asset index								
1st quintile	62 (77.50)	18 (22.50)	1	-	**<0.0001**	1	-	0.927
2nd quintile	60 (80.00)	15 (20.00)	0.88	0.48–1.63		0.91	0.49–1.69	
3rd quintile	44 (68.75)	20 (31.25)	1.38	0.80–2.39		0.9	0.51–1.60	
4th quintile	54 (65.85)	28 (34.15)	1.51	0.91–2.51		0.93	0.51–1.70	
5th quintile	23 (35.94)	41 (64.06)	**2.84**	**1.82–4.45**		1.07	0.58–1.94	
PN visits**								
0–5	65 (81.25)	15 (18.75)	1	-	**<0.0001**	1	-	**0.05**
6–9	153 (71.16)	62 (28.84)	1.53	0.93–2.54		1.82	0.86–3.87	
10 or more	78 (46.99)	88 (53.01)	**2.82**	**1.75–4.56**		**2.33**	**1.10–4.96**	
Mother’s age								
<=20	193 (77.20)	57 (22.80)	1	-	**<0.0001**	1		0.051
21–34	110 (49.77)	111 (50.23)	**2.2**	**1.69–2.86**		**1.67**	**1.07–2.60**	
35 or more	3 (33.33)	6 (66.67)	**2.92**	**1.74–4.89**		1.27	0.59–2.74	
Seen by SUS***								
Yes	25 (25.00)	75 (75.00)	1	-	**<0.0001**	1	-	**<0.001**
No	281 (73.95)	99 (26.05)	**2.87**	**2.34–3.54**		**2.27**	**1.44–3.60**	
Mother lives with the husband or partner								
Yes	78 (69.64)	34 (30.36)	1	-	0.153	1	-	0.992
No	228(61.96)	140 (38.04)	1.25	0.91–1.70		0.99	0.66–1.48	
Mother’s skin color								
White	218 (61.58)	136 (38.42)	1	-	0.261	-	-	-
Black	68 (70.83)	28 (29.17)	0.75	0.54–1.06				
Other	20 (66.67)	10 (33.33)	0.86	0.51–1.46				

*95% confidence interval

**Prenatal

***Public Healthcare System

Given the probability of interaction between the variables of having delivery by SUS and the number of prenatal consultations, a heterogeneity test was performed, which showed no interaction between them (*p*-value = 0.222).

After the multivariate analysis, the risk factors for repeat cesarean section included ages 21–34 (PR 1.67, CI 95% 1.07–2.60), not being seen by SUS in 2004 (PR 2.27, CI 95% 1.44–3.60), and the number of prenatal medical visits, i.e., women with ten or more visits were at 2.33 times higher risk (CI 95% 1.10–4.96) compared to those who had five or fewer visits (Table 2).

## Discussion

The results in the present study point to the influence of factors of socioeconomic nature and related to prenatal care and childbirth on the recurrence of surgical delivery in a subsequent pregnancy among primiparous women who underwent a cesarean section. Repeat cesarean sections were positively associated with the AI, schooling, mother’s age, with a higher number of prenatal medical visits and being seen by healthcare services other than SUS.

In the present study, repeat cesarean sections were associated with the number of prenatal medical visits and the risk was higher among those with more visits. However, a prenatal visit must be used to educate the mother-to-be regarding the benefits and risks of each mode of delivery and not a risk factor for having cesarean sections as it was found to be. Instead of soothing the fear and insecurity that every pregnant woman feels, prenatal care ends up stimulating such feelings [14].

In an ideal healthcare scenario, the higher risk of repeat cesarean sections among women who attend more prenatal medical visits could indicate those women were at greater gestational risk. However, several Brazilian studies have shown inequities in prenatal care by evidencing that younger women with lower income and no access to private health insurances – who, therefore, would be at higher obstetrical risk – are more likely to receive inappropriate prenatal care [14, 15]. Prenatal coverage also progressively increases with family income, which means these healthcare services expand the differences that discriminate poorer women instead of correcting them [16].

A study carried out on pregnant Brazilian adolescents also reported that a higher number of prenatal medical visits increased the odds of a cesarean section. This finding is probably because physicians who see the expecting patients more times are more likely to convince the women of their preferred mode of delivery, particularly when the same professional will assist the birth [17].

The large percentage of cesarean sections found among this population matches the high rates of these deliveries in the country as a whole, which are among the highest worldwide [4, 5] regardless of the governmental recommendations and attempts to lower them [18, 19].

A recent Brazilian population-based study pointed to a growing preference for cesarean section among women, reportedly at almost a third of them. However, significant differences were found according to the reproductive background and source of labor funding, with lower rates (15.4%) among nulliparous in the public healthcare and higher rates (73.2%) among multiparae with previous cesarean section in the private healthcare [20].

Although a meta-analysis has already shown that vaginal delivery after cesarean section is safe [8], only 12.56% of the women who underwent a cesarean section in the first delivery had a vaginal delivery in the subsequent pregnancy, similarly to what was found in another Brazilian study that showed that only 14.8% of the women with a previous cesarean section had a vaginal delivery and, of those, 62% underwent a Cesarean section without labor [20]. The prevalence of vaginal delivery after trial of labor is significantly high, at 74% in the United States [11].

Besides offering an option to women who want to experience vaginal delivery, this mode after a cesarean section has potential advantages to the women’s health, who avoid an extensive abdominal surgery, have lower rates of blood transfusion and hysterectomy, a shorter recovery period, and avoid all the other complications associated with cesarean sections compared to women who undergo a repeat cesarean section [21, 22]. The risk of placental accreta, cystotomy, intestinal, urethral, and ileum lesions, and the need for ventilation support, admission in intensive care unit, hysterectomy, and blood transfusion, as well as the risk of a longer hospitalization stay significantly increases with a higher number of cesarean section deliveries [23].

The successive cesarean sections put the women at higher risk of obstetrical and postpartum complications. Women with multiple cesarean sections are at higher risk of large adherences, intestinal and bladder lesions, abnormal placental implantation, hysterectomy, blood transfusion, and intensive care unit admission [24], which shows this procedure is not free of risk and that it must, therefore, have clear and precise medical indications.

In the present study, the true indications for cesarean sections could not be assessed either in the first delivery or in the subsequent one, therefore, this type of analysis cannot be made. However, it is believed that many of those women had no clear, precise clinical indications to the surgical procedure since the percentage of cesarean sections was significantly higher.

A previous study with the same sample attempted to identify which cesarean sections were elective, however, such information could not be obtained in the medical records because the physicians are reluctant to admit the surgery had no clinical indication [25].

The high percentage of repetition of the way of delivery, whether cesarean or vaginal, makes it clear that cesarean should be avoided, whenever possible, in the first gestation. This requires a great change in culture and in the Brazilian healthcare system. The cultural change involves informational campaigns that make it clear that a cesarean section does not represent better quality of care or absence of pain, and that vaginal delivery does not interfere with sexual pleasure [26].

Changes in the healthcare system and obstetric care are more complex. Individualized healthcare, centered around the doctor, favors unnecessary caesarean sections, especially when the same professional is in charge of prenatal care and delivery. Childbirth care should be provided by a healthcare team and the presence of the midwife or obstetrician nurse should be reinstated.

## Conclusions

This study on a cohort of Brazilian women showed a quite high proportion of cesarean sections both in the first gestation and in subsequent ones. Repeat cesarean sections were associated with mother’s age, number of prenatal visits, and delivery carried out by healthcare providers out of the public healthcare system, SUS.

Repeat cesarean sections may lead to increased risk of obstetrical complications such as large adherences, intestinal and bladder lesions, abnormal placental implantation, hysterectomy, blood transfusion, and intensive care unit admission [24], which may impact the reproductive future of those women.

Effective changes in obstetric care must be implemented to point out the benefits of vaginal delivery for both the woman and the child. Maternal health care providers should be trained to provide respectful and individualized care to the mother and the neonate, thus ensuring the safety of both during birth. Effective public policies that ensure the continuity of care during gestation and birth are also important and may help reduce cesarean section rates either in the first gestation or in subsequent ones.

## Additional file

Additional file 1: Ethics committee in research. (DOCX 1479 kb)

## Abbreviations

AI: Asset index
SUS: Public Healthcare System
