# Supplementary figures and images for: Repeat cesarean section in subsequent gestation of women from a birth cohort in Brazil
Source: Reprod Health. 2017 Aug 25;14:102. doi: 10.1186/s12978-017-0356-8 (PMC5572067; doi:10.1186/s12978-017-0356-8)

**Perinatal and 3 months**

- **48 months**

**
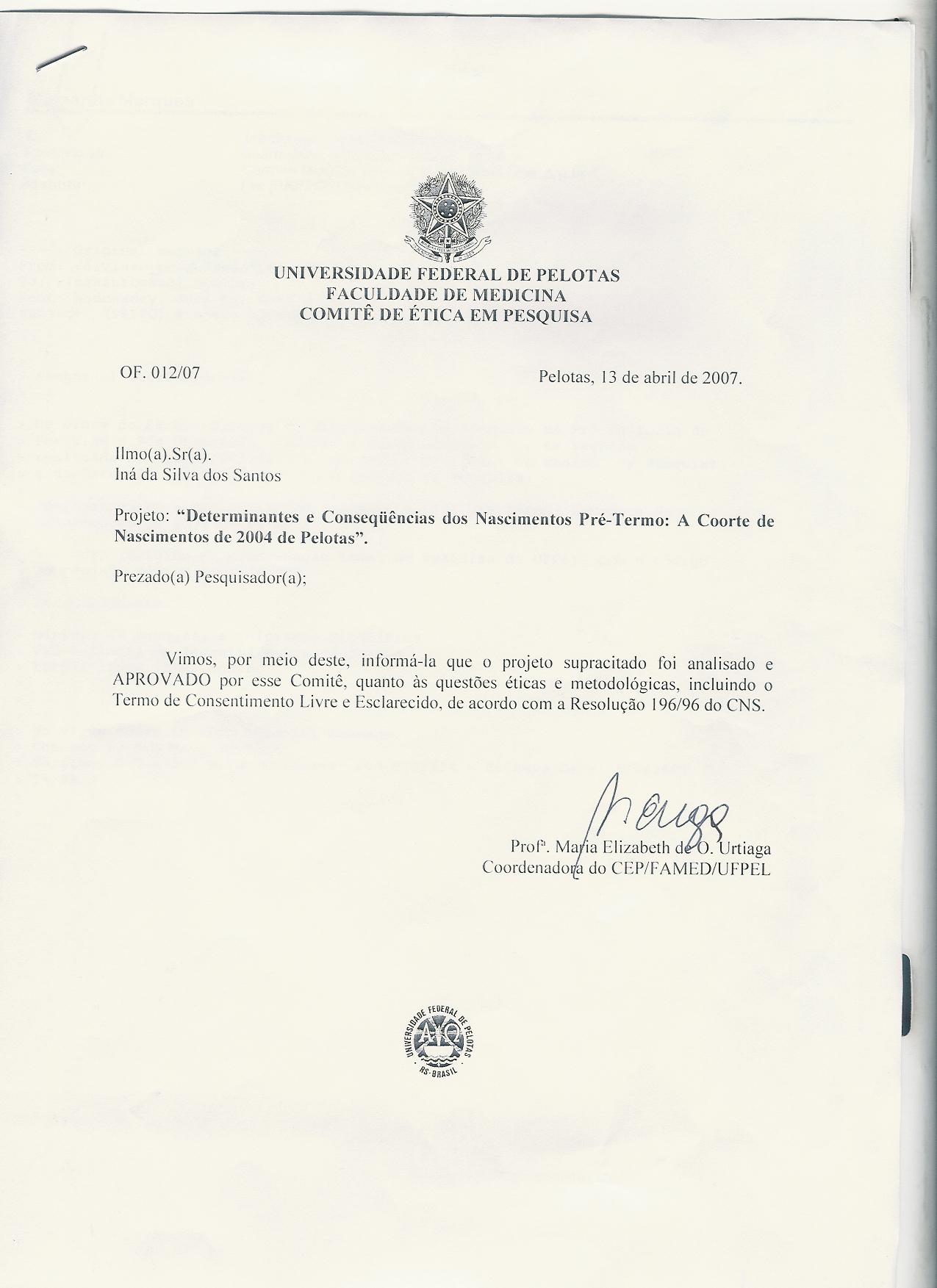
**

**24 months**

**
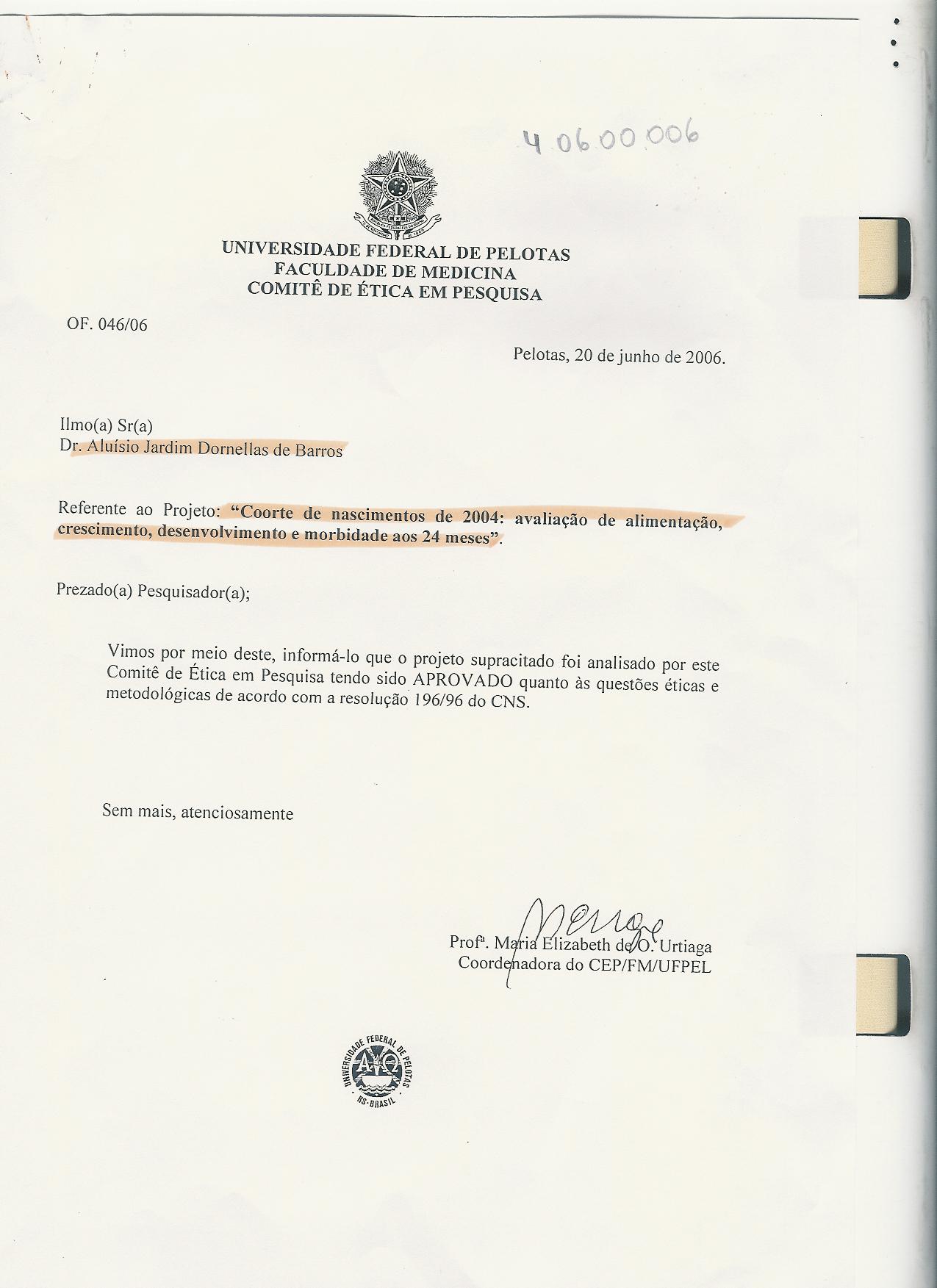
**

**12 months**

**
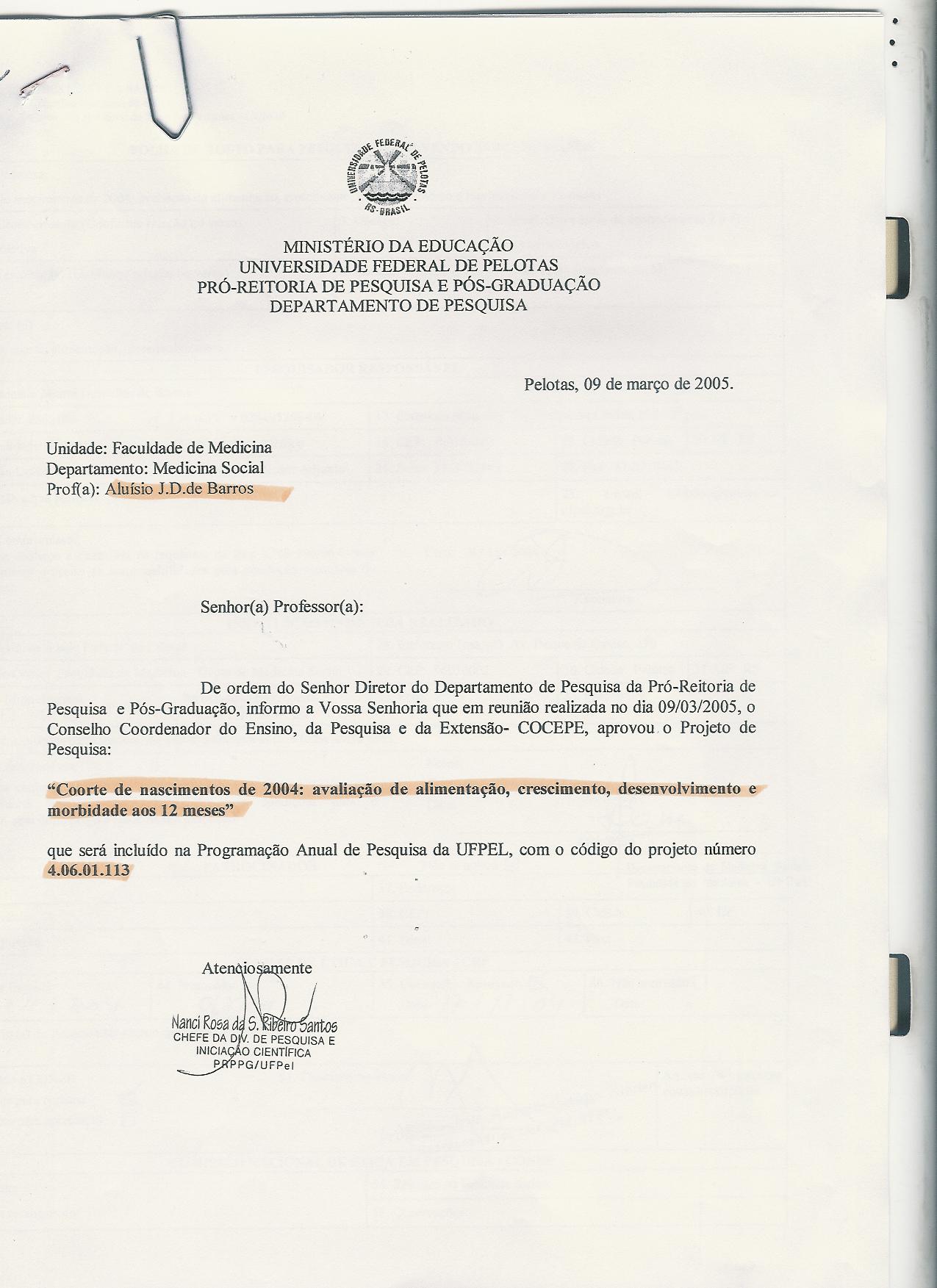
**

**6-7 years**

**
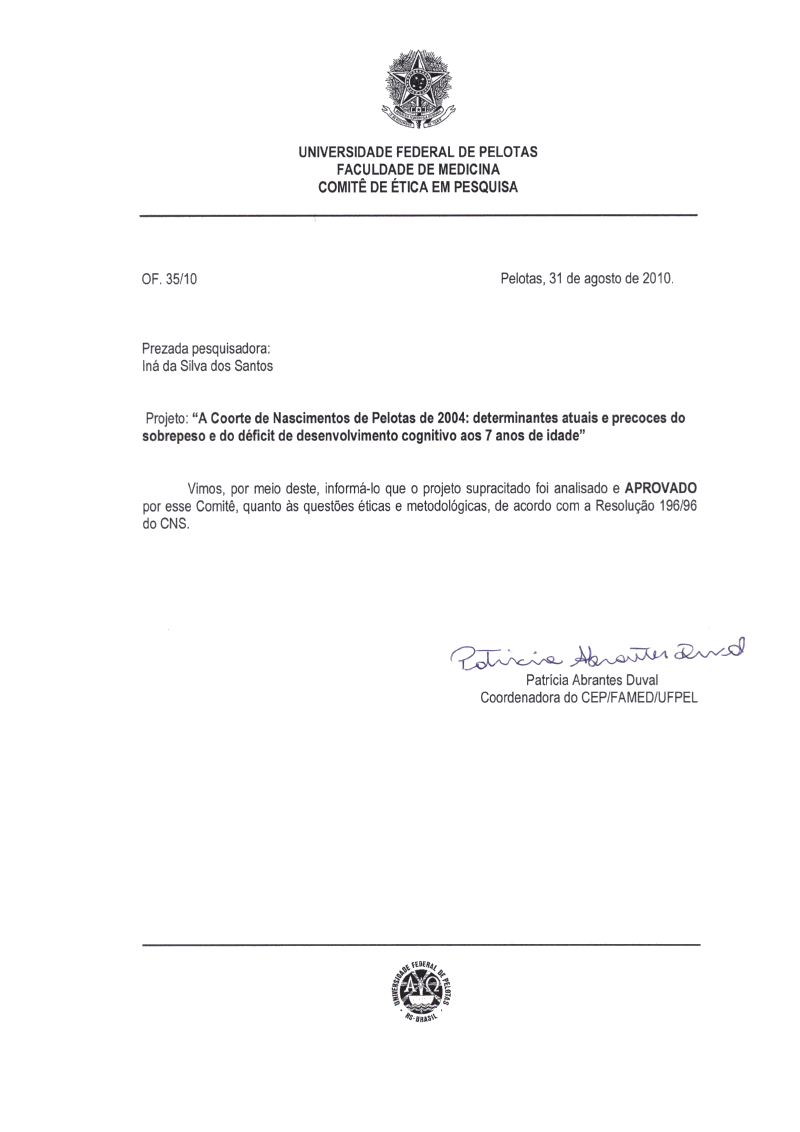
**

Supplement: Additional file 1: — Ethics committee in research. (DOCX 1479 kb) [file 12978_2017_356_MOESM1_ESM.docx]
